# Identification of Key Candidate Proteins and Pathways Associated with Temozolomide Resistance in Glioblastoma Based on Subcellular Proteomics and Bioinformatical Analysis

**DOI:** 10.1155/2018/5238760

**Published:** 2018-03-01

**Authors:** Guo-zhong Yi, Wei Xiang, Wen-yan Feng, Zi-yang Chen, Yao-min Li, Sheng-ze Deng, Man-lan Guo, Liang Zhao, Xue-gang Sun, Min-yi He, Song-tao Qi, Ya-wei Liu

**Affiliations:** ^1^Department of Neurosurgery, Nanfang Hospital, Southern Medical University, Guangzhou, Guangdong 510515, China; ^2^The Laboratory for Precision Neurosurgery, Nanfang Hospital, Southern Medical University, Guangzhou, Guangdong 510515, China; ^3^Department of Neurosurgery, The First Affiliated Hospital, Southwest Medical University, Luzhou, Sichuan 646000, China; ^4^Department of General Intensive Care Unit, The Third Affiliated Hospital of Sun Yat-sen University of Medical Sciences, Guangzhou 510630, China; ^5^Guangdong Provincial Key Laboratory of Molecular Oncologic Pathology, Guangzhou, Guangdong 510515, China; ^6^School of Traditional Chinese Medicine, Southern Medical University, Guangzhou, Guangdong 510515, China; ^7^Center for Clinical Medical Education, Nanfang Hospital, Southern Medical University, Guangzhou, Guangdong 510515, China; ^8^Nanfang Glioma Center, Guangzhou, Guangdong 510515, China

## Abstract

TMZ resistance remains one of the main reasons why treatment of glioblastoma (GBM) fails. In order to investigate the underlying proteins and pathways associated with TMZ resistance, we conducted a cytoplasmic proteome research of U87 cells treated with TMZ for 1 week, followed by differentially expressed proteins (DEPs) screening, KEGG pathway analysis, protein–protein interaction (PPI) network construction, and validation of key candidate proteins in TCGA dataset. A total of 161 DEPs including 65 upregulated proteins and 96 downregulated proteins were identified. Upregulated DEPs were mainly related to regulation in actin cytoskeleton, focal adhesion, and phagosome and PI3K-AKT signaling pathways which were consistent with our previous studies. Further, the most significant module consisted of 28 downregulated proteins that were filtered from the PPI network, and 9 proteins (DHX9, HNRNPR, RPL3, HNRNPA3, SF1, DDX5, EIF5B, BTF3, and RPL8) among them were identified as the key candidate proteins, which were significantly associated with prognosis of GBM patients and mainly involved in ribosome and spliceosome pathway. Taking the above into consideration, we firstly identified candidate proteins and pathways associated with TMZ resistance in GBM using proteomics and bioinformatic analysis, and these proteins could be potential biomarkers for prevention or prediction of TMZ resistance in the future.

## 1. Introduction

Glioblastoma (GBM) remains one of the most lethal cancers for human beings and the prognosis of GBM is still pessimistic [1, 2]. The treatment of GBM includes gross total resection of tumor tissues, followed by adjuvant chemo- and radiotherapy. Temozolomide (TMZ) is the first-line chemotherapy agent and has improved the prognosis of GBM patients significantly [3, 4]. However, development of TMZ resistance during therapy period is so common among clinical GBM patients, which is also one of the main causes why treatment fails. The most popular mechanism of TMZ resistance is the expression of O6-methylguanine-DNA methyltransferase (MGMT) which protects the cellular genome from the damage of alkylating agents [5]. However, for the acquired TMZ resistance of GBM cells, the mechanism still remains unclear.

Over the past years, extensive genomics and proteomics studies have been conducted and also greatly advanced our understanding of the molecular mechanism which underlies the pathogenesis of GBM. However, most of these studies mainly focus on the whole proteome and genome. Subcellular distribution of proteins under different conditions is a major challenge in cell biology indeed; thus subcellular proteome has been developed to address this issue reliably [6]. Simultaneously, using bioinformatic analysis such as gene ontology (GO), pathway enrichment information, and network-based approaches can also help us to identify the exact molecular mechanism.

Our previous researches indicated that TMZ treatment for 1 week could induce the reconstruction of cytoskeleton [7] and activate epithelial-to-mesenchymal transition (EMT) and autophagy process of GBM cells [8]. And phosphoinositide 3-kinase (PI3K) signaling pathway also contributed to the acquired TMZ resistance of GBM cells [9]. In order to explore key candidate proteins and pathways which may play important roles in acquired TMZ resistance, and in consideration of all the biological processes identified in our previous studies occurring in cytoplasma, then we conducted a cytoplasmic proteome research of U87 GBM cells treated with TMZ for 1 week. Further we performed KEGG pathway enrichment analysis, protein–protein interaction (PPI) network and subnetwork analysis, and validation of key candidate proteins in TCGA-GBM dataset. Finally 9 important proteins were identified as the key candidate proteins, which may serve as reliable biomarkers for the prediction or prevention of acquired TMZ resistance of GBM in the future.

## 2. Materials and Methods

### 2.1. Cell Culture and Protein Extraction

The human glioblastoma U87 cell line was purchased from the American type culture collection (ATCC, USA) and cultivated in Dulbecco's modified Eagle's medium (DMEM; Gibco, Grand Island, NY) supplemented with 10% FBS and cultured at 37°C in a humidified atmosphere of 5% CO2. TMZ was purchased from Sigma-Aldrich (St. Louis, MO, USA) and diluted in dimethyl sulfoxide (DMSO) (Solarbio Inc., Beijing, China) to a stock solution of 200 mM TMZ. Immediately before use in cell culture, the stock was diluted in media to a concentration of 200 *μ*M. The same concentration of DMSO was added to the control group.

The method of trypan blue staining was described in our previous study [7]. Nuclear and cytoplasmic fractions were generated according to the protocol as follows: 2 × 10^7^ cells were collected and suspended in 300–500 *μ*l RLN lysis buffer (Qiagen, Germany) with 1% PMSF, kept on ice for 20 min, and centrifuged for 10 min at 3000 ×g, 4°C; the clear supernatant was the cytoplasmic extractions; the nucleus precipitate was washed with RLN lysis buffer 3 times and the supernatant was discarded; the nucleus precipitate was suspended in 100 *μ*l RIPA lysis buffer (Cell Signaling Technology, USA) with 1% PMSF, with sonication on ice for 1 min; the suspension was centrifuged at 12000 ×g for 20 min at 4°C; the clear supernatant was the nuclear extractions. The validation of purity of the extractions was indicated for western blots probed with antibodies against two fractionation controls (Histone H3 for nucleus extraction, Beyotime, China; dilution 1 : 1000; GAPDH for cytoplasma extraction, Jetway Biotech Co., Ltd., China; dilution 1 : 2000).

### 2.2. Protein Digestion and Liquid Chromatography–Electrospray Ionization–Tandem Mass Spectrometry (LC–ESI–MS/MS)

Digestion of protein (250 *μ*g for each sample) was performed according to the FASP procedure described by Wisniewski [10]. The resultant peptides of trypsin digestion were desalted by solid phase extraction using C_18_ Empore disc cartridges (Supelco/Sigma-Aldrich, Taufkirchen, Germany) [11] and then concentrated by vacuum centrifugation and reconstituted in 40 *μ*l of 0.1% trifluoroacetic acid. MS experiments were performed on a Q Exactive mass spectrometer that was coupled to Easy nLC (Proxeon Biosystems, Thermo Fisher Scientific, Bremen, Germany). 5 *μ*g peptide was loaded onto a the C_18_-reversed phase column (Thermo Scientific, Easy Column, 10 cm long, 75 *μ*m inner diameter, and 3 *μ*m resin) in buffer A (2% acetonitrile and 0.1% formic acid) and separated with a linear gradient of buffer B (80% acetonitrile and 0.1% formic acid) at a flow rate of 250 nL/min controlled by IntelliFlow technology over 120 min. MS data was acquired using a data-dependent top-10 method dynamically choosing the most abundant precursor ions from the survey scan (300–1800* m/z*) for HCD fragmentation. Determination of the target value is based on predictive Automatic Gain Control (pAGC). Dynamic exclusion duration was 25 s. Survey scans were acquired at a resolution of 70,000 at* m/z* 200 and resolution for HCD spectra was set to 17,500 at* m/z* 200. Normalized collision energy was 30 eV and the underfill ratio, which specifies the minimum percentage of the target value likely to be reached at maximum fill time, was defined as 0.1%. The instrument was run with peptide recognition mode enabled. MS experiments were performed triply for three biologic repetitions.

### 2.3. Protein Identification

The raw MS/MS spectra search was carried out using a freely available software suite, MaxQuant (version. 1.3.0.5). MS data were searched through the UniProtKB database [12]. An initial search was set at a precursor mass window of 6 ppm. The search followed an enzymatic cleavage rule of trypsin and allowed maximal two missed cleavage sites and a mass tolerance of 20 ppm for fragment ions. Carbamidomethylation of cysteines was defined as fixed modification, while protein N-terminal acetylation and methionine oxidation were defined as variable modifications for database searching. The cutoff of global false discovery rate (FDR) for peptide and protein identification was set to 0.01. The MaxLFQ label-free quantification method, a retention time alignment and identification transfer protocol (“match-between-runs” feature in MaxQuant) described in [13], was applied and a novel algorithm was used to extract the maximum possible quantification information. Protein abundance was calculated on the basis of the normalized spectral protein intensity (LFQ intensity). The mass spectrometry proteomics data have been deposited to the ProteomeXchange Consortium via the PRIDE partner repository with the dataset identifier PXD007759.

### 2.4. Identification of Significantly Differently Expressed Proteins (DEPs), Gene Ontology, and Pathway Enrichment Analysis

The raw data of cytoplasmic proteome expression was analyzed. Differently expressed proteins (DEPs) were identified with classic Student's *t*-test, and the log_2_ fold change (log_2_FC) was also calculated. |log_2_⁡FC| ≥ 1 and *P* value < 0.05 were considered as the cutoff values for DEPs screening. We did not apply adjusting for multiple hypothesis testing for DEPs screening. Candidate DEPs functions and pathways enrichment were analyzed using online databases. The Database for Annotation, Visualization, and Integrated Discovery (DAVID) was a comprehensive set of functional annotation tools. We performed GO and KEGG pathway enrichment analyses using the DAVID online tool (https://david.ncifcrf.gov/) and KEGG pathway tool (http://www.genome.jp/kegg). *P* value < 0.05 was set as the cutoff criterion.

### 2.5. Protein–Protein Interaction (PPI) Network Construction, Modular Analysis, and Significant Candidate Proteins and Pathway Identification

Online database Search Tool for the Retrieval of Interacting Genes (STRING, https://string-db.org) [14] was applied to analyze the protein–protein interaction (PPI) network. Cytoscape software [15] was utilized to construct protein interaction relationship network and analyze the interaction relationship of the candidate DEPs. Each node represented a protein and the edges represented the interactions between proteins. The Network Analyzer plug-in was used to calculate node degree, which means the numbers of interconnections, and to filter the key candidate proteins of PPI with degree ≥12. These proteins might be the core proteins and may have important physiological regulatory functions.

For validation of the significant candidate DEPs, The Cancer Genome Atlas (TCGA) Glioblastoma dataset was analyzed using R2: Genomics Analysis and Visualization Platform (http://hgserver1.amc.nl/cgi-bin/r2/main.cgi). The TCGA Glioblastoma dataset included 540 samples.

## 3. Results

### 3.1. Global Profiling of Cytoplasmic Proteins in U87 Cells by Label-Free Quantitative Proteomics

**Figure 1:**
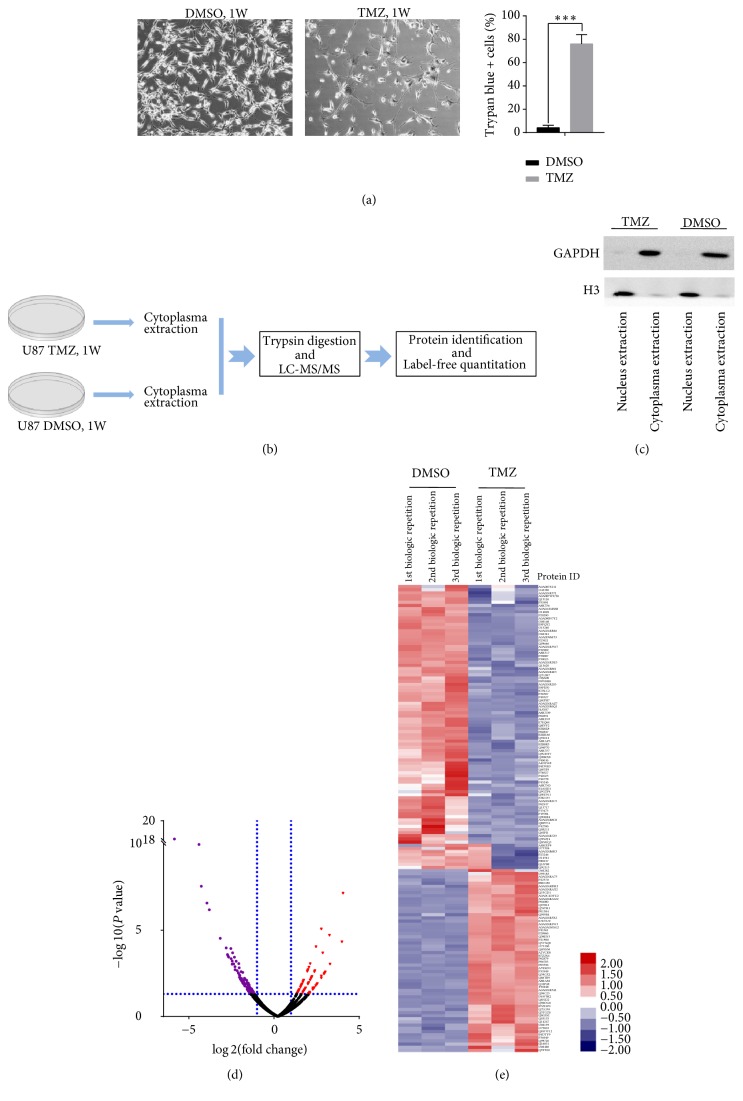
Cytoplasmic proteome research of U87 GBM cells treated with TMZ for 1 week. (a) Morphology and viability change of U87 cells after temozolomide treatment for 1 week; (b) outline of the experimental workflow; (c) verification of the purity of nucleus and cytoplasma extractions; (d) volcano figure of all identified proteins. Red plots mean upregulated proteins in cytoplasma after TMZ treatment, and purple plots mean downregulated proteins. |log⁡2FC|⩾1 and *P* value < 0.05 were considered as the cutoff values for DEPs screening; (e) Heat map of the significant DEPs.

We treated U87 cells with 200 *μ*M TMZ for 1 week; only 20% of the cells survived and showed great phenotypic changes (Figure 1(a)). Then we extracted the cytoplasmic lysates as the proteome samples; the flowchart was shown in Figure 1(b). The purity and efficiency of nucleus and cytoplasma extraction were verified through western blot (Figure 1(c)). Protein identification and label-free quantification were carried out by analyzing MS raw data using the MaxQuant-Andromeda software suite. Finally, it revealed a total of 1999 nonredundant proteins based on the identification of one or more unique peptides. The mass spectrometry proteomics data have been deposited to the ProteomeXchange Consortium via the PRIDE partner repository with the dataset identifier PXD007759.

### 3.2. Identification of Differentially Expressed Proteins (DEPs)

A total of 161 proteins displayed more than 2-fold quantitative alterations with *P* value < 0.05 in the proteome comparison between TMZ-treated group and DMSO-treated group, including 65 upregulated proteins (1 protein identified as* Bos taurus* species was excluded) and 96 downregulated proteins (Figures 1(d) and 1(e) and Table 1).

### 3.3. Gene Ontology Analysis and Signaling Pathway Enrichment Analysis

**Figure 2:**
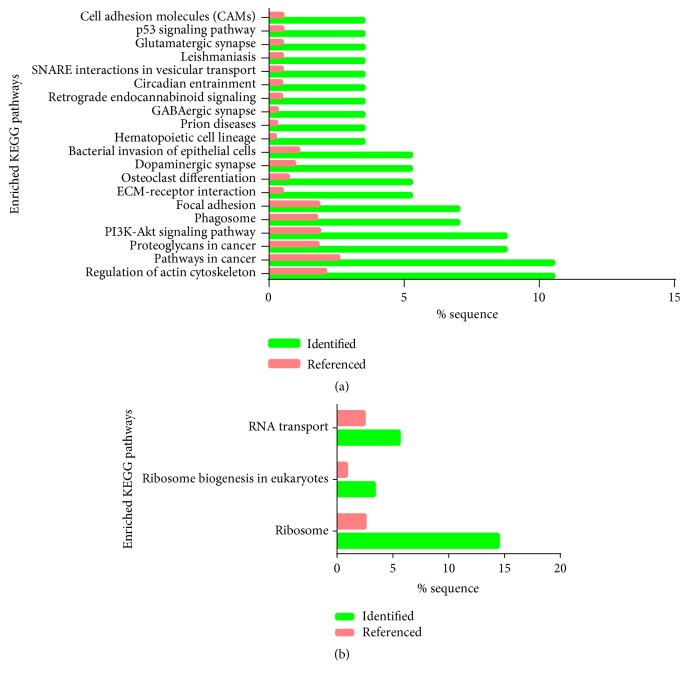
Signaling pathway enrichment analysis of DEPs identified in this research. Significantly enriched KEGG terms of upregulated DEPs (a) and downregulated DEPs (b), identified bar represented the percentage of DEPs enriched in specific KEGG term, and the referenced bar represented the percentage of all identified proteins enriched in this KEGG term, which act as a control.

Proteins were subjected to signaling pathway enrichment analyses; the pathways enriched by downregulated DEPs were mainly related to ribosome and RNA transport (Figure 2(b) and Table 2). The pathways enriched by upregulated DEPs were mainly related to regulation in actin cytoskeleton, pathways in cancer, focal adhesion, and phagosome and PI3K-AKT signaling pathway (Figure 2(a) and Table 2).

### 3.4. Key Candidate Proteins Identification with Protein–Protein Interaction (PPI) Network and Subnetwork Analysis

**Figure 3:**
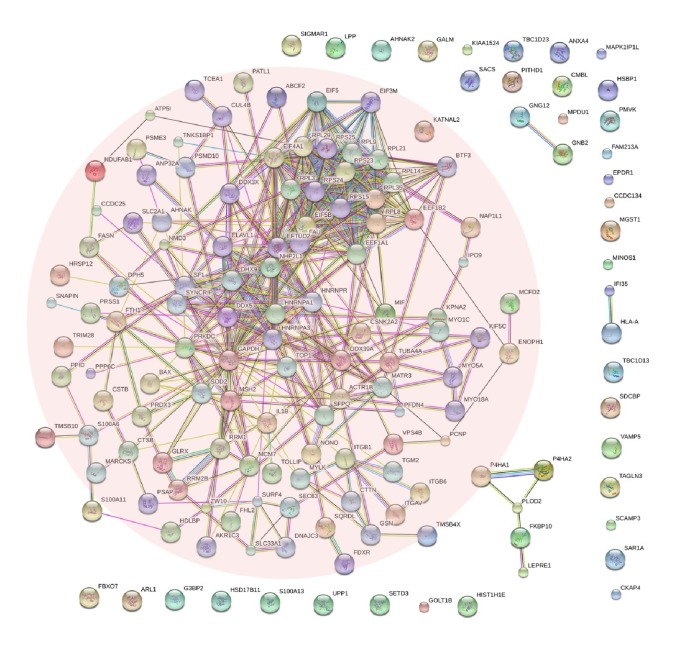
Protein–protein interaction (PPI) network analysis of DEPs. Based on STRING online database, a total of 161 DEPs constructed the PPI network. The nodes included in the pink region had strong interactions with other nodes, which indicated significant importance for the screening of key candidate proteins.

**Figure 4:**
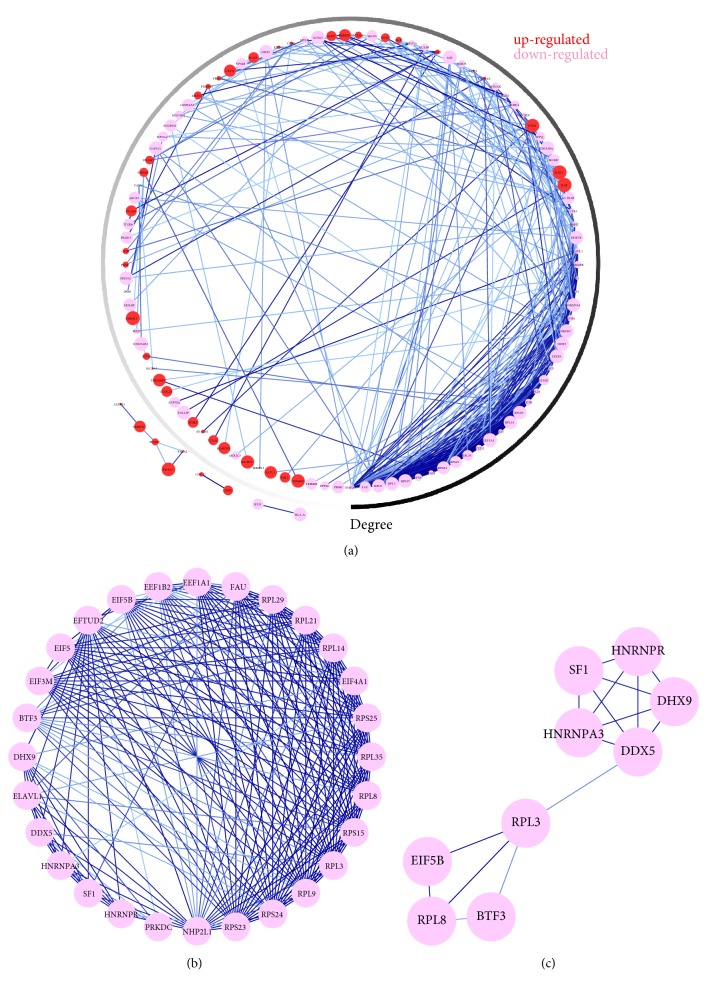
Protein–protein interaction (PPI) network analysis of DEPs. (a) Subnetwork analysis of DEPs for key candidate proteins identification; (b) a total of 28 key candidate proteins whose degree *⩾*12 were identified in this study, and this module consisted of 28 nodes and 452 edges; (c) this module consisted of 9 special key candidate proteins with significant relation with prognosis of GBM patients, which were mainly associated with ribosome and spliceosome signaling pathway. Red nodes represented the upregulated DEPs and pink nodes represented downregulated DEPs, the size of each node was determined by the significance of *P* value (larger size means more significant), the color of outer circle represented the numbers of degrees (the largest was 27 and the smallest was 1), and the deeper color of the edge represented a higher combined score between two nodes.

Using the STRING online database (https://string-db.org) (Figure 3) and Cytoscape software, the PPI network was constructed (Figure 4(a)). A total of 161 DEPs (65 upregulated and 96 downregulated proteins) were included in the PPI network; there existed 2 subgroups: proteins with strong connections with others (the pink region shown in Figure 3) and separated proteins. So we conducted subnetwork analysis among these proteins which have strong interactions with each other. As shown in Figure 4(a), the subnetwork contained 114 nodes (44 upregulated and 70 downregulated proteins) and 822 edges. In order to identify key candidate proteins related to TMZ therapy, we selected 28 hub proteins with the filtering of degree ≥12 criteria (i.e., each node had more than 12 connections/interactions) (Figures 4(a) and 4(b)); all these 28 proteins were downregulated proteins and mainly associated with ribosome pathway and spliceosome pathway.

### 3.5. Validation of the Key Candidate Proteins in TCGA Database

**Figure 5:**
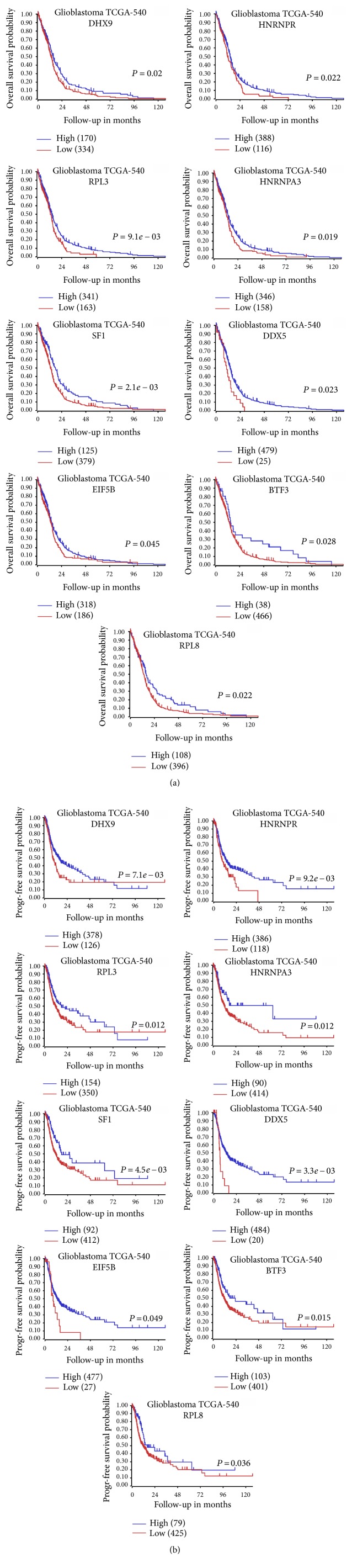
Validation of the key candidate proteins in TCGA-GBM-540 dataset (a total of 504 patients; 36 patients lacking survival data were omitted from the analysis). Nine special proteins (DHX9, HNRNPR, RPL3, HNRNPA3, SF1, DDX5, EIF5B, BTF3, and RPL8), which were downregulated after TMZ treatment and related to both overall survival (a) and progress-free survival (b) probability of GBM patients.

To validate the reliability of the 28 key candidate proteins, we used R2 Platform to confirm the predictive capability for overall survival or progress-free survival probability in TCGA 540 GBM dataset. We found that all the 28 key candidate proteins (Figure 4(b)) were significantly related to the prognosis of GBM patients (either OS or PFS), suggesting our results of the identified candidate proteins were reliable (data not shown). Among the 28 key candidate proteins, we also found 9 special proteins (DHX9, HNRNPR, RPL3, HNRNPA3, SF1, DDX5, EIF5B, BTF3, and RPL8) (Figure 4(c)), which were downregulated after TMZ treatment, and lower expression indicated worse prognosis of GBM patients (both OS and PFS) (Figure 5). Among them, RPL3, RPL8, BTF3, and EIF5B were associated with ribosome signaling pathway and the other 5 proteins were related to spliceosome signaling pathway.

## 4. Discussion

Although the use of TMZ has improved the OS of GBM patients from 12.6 to 14.6 months [1], the development of drug resistance is still one of the main causes of treatment failure, and the molecular mechanism of this drug resistance phenomenon still remains unclear. In this study, we conducted a cytoplasmic proteome research of U87 GBM cells treated with TMZ for 1 week, utilized bioinformatic methods to analyze the raw data deeply, and identified 161 significant DEPs including 65 upregulated and 96 downregulated proteins.

The upregulated DEPs were mainly enriched in regulation of actin cytoskeleton, pathways in cancer, PI3K-Akt pathway, and phagosome and focal adhesion signaling pathways. Consistent with our previous researches, we have already found that treatment of TMZ in U87 GBM cells could induce reconstruction of cytoskeleton [7], EMT-like changes, and activation of autophagy process [8], which would protect cells from the nucleus damage induced by TMZ. Further, we have also found that PI3K-Akt pathway was activated in acquired TMZ-resistant U87 cells, which may contribute to the chemoresistance [9]. The downregulated DEPs were mainly enriched in ribosome and RNA transport pathway.

In order to screen the key candidate proteins responsible for TMZ resistance, we conducted PPI-network analysis, and 28 DEPs were identified finally. Interestingly, all these 28 proteins were downregulated DEPs, and all of them were related to the prognosis of GBM patients (either OS or PFS). After further investigating and validating TCGA-GBM dataset, a total of 9 proteins (DHX9, HNRNPR, RPL3, HNRNPA3, SF1, DDX5, EIF5B, BTF3, and RPL8) among them were identified as the key candidate proteins which were related to TMZ therapy. All these 9 proteins were downregulated in the cytoplasma of TMZ-treated U87 cells, and lower expression of these proteins indicated worse prognosis of GBM patients (both OS and PFS) after analyzing the TCGA-GBM-540 database. For the survival analysis, the results could be more robust if we have done a survival analysis of subgroup only including GBM cases treated with TMZ. All these 9 proteins identified in this study were significantly associated with ribosome and spliceosome signaling pathways.

Ribosome, consisting of nucleic acids and proteins, catalyzes protein synthesis according to the genetic instructions in all organisms [16], and ribosomal associated proteins interconnect ribosome with diverse cellular processes, providing an additional layer of regulatory potential to protein expression [17]. EIF5B, a conserved eukaryotic translation initiation factor, is an identified key protein in this study. Factors EIF1A and EIF5B interact on the ribosome to position the initiation methionine tRNA on the start codon of the mRNA translation so that translation initiates accurately [18]. BTF3 as the basic transcription factor is also required for transcriptional initiation [19], and previous research has also revealed that BTF3 was downregulated in GBM which is consistent with our study [20]. In our study, RPL3 and RPL8 were identified as dysregulated ribosomal associated proteins of U87 cells. RPL8, a component of the 60S subunit of ribosome, is involved in protein synthesis and RPL8 antigen may represent a relevant vaccine target for patients with glioma [21]. Besides, the restoration of RPL3 protein may resensitize the resistant cells to the drug by regulating the reactive oxygen species (ROS) levels in lung cancer cells [22]. The nucleolus is the site of ribosome biogenesis and ribosomal proteins also play an important role in the response to nucleolar stress, as TMZ can induce DNA damage seriously [23]. Recently, Fancello and colleagues have identified inactivating mutations and deletions in RPL5 in 10% of GBM cases and showed that these lesions are associated with worse outcome in GBM [24]. Due to the stoichiometry of the ribosome, expression of ribosomal proteins is tightly connected and loss of expression of one component can reduce expression of the entire ribosome. One suppositional mechanism may be that the lesions of RPL5 downregulate the other components of the ribosome observed in this study (RPL3 and RPL8). However, this needs to be confirmed in the future.

Alternative splicing of mRNA precursors enables one gene to produce multiple protein isoforms with differing functions, and aberrant splicing of mRNA precursors leads to production of aberrant proteins that contribute to tumorigenesis [25, 26]. Another pathway enriched in these key candidate proteins was spliceosome signaling pathway. These proteins were as follows: SF-1 which is a nuclear pre-mRNA splicing factor [27], DDX5 which is involved in nuclear and mitochondrial splicing and ribosome and spliceosome assembly [28], HNRNPR which is a member of the spliceosome C complex and function in pre-mRNA processing and transport [29], and HNRNPA3 which is found to interact with SOX2 and HNRNPR and play a possible role in posttranscriptional regulation [30]. Notably, there exists alternative splicing process in the synthesis of DHX9. For DHX9, treatment with chemotherapeutic drugs such as etoposide could elicit DHX9 splicing, and the new isoform of the RNA helicase DHX9 has great impacts on the regulation of cell responses to DNA damage [31, 32]. Thus, the dysregulation of these spliceosome related proteins may jointly contribute to a disorder of the synthesis of DHX9 and result in the TMZ resistance of GBM ultimately. However, the mechanism still needs verification of further researches and experiments.

## 5. Conclusion

We have identified 161 DEPs related to TMZ therapy in GBM through cytoplasmic proteome research and finally found 9 mostly changed hub proteins which were significantly enriched in ribosome and spliceosome signaling pathway after performing further bioinformatics analysis. These findings of our study may contribute to the understanding of the underlying molecular mechanisms of TMZ resistance in GBM cells, and the candidate proteins and pathways can be used as potential biomarkers for the prevention or prediction of TMZ resistance in the future.

## Figures and Tables

**Table 1:** List of 161 DEPs identified in the proteome analysis, including 65 upregulated proteins and 96 downregulated proteins in cytoplasma of U87 GBM cells treated with TMZ compared to the control group treated with DMSO.

DEPs	Protein name
Upregulated	CCDC25, HIST1H1E, KIF5C, SNAPIN, VAMP5, SLC33A1, GOLT1B, IL1B, PCNP, MINOS1, ZW10, UPP1, CTTN, FDXR, TOP1, RRM2B, ANXA4, PSMD10, ITGB, CKAP4, S100A13, LPP, ATP5I, TCEA1, TRG14, SLC2A1, TUBA4A, GNG12, hCG, CTSB, MYO1C, SIGMAR1, MAPK1IP1L, MGST1, SEC22B, ITGB1, P4HA2, FTH1, CSTB, ITGAV, GLRX, BAX, PRDX3, PLOD2, DNAJC3, PSAP, S100A11, LEPRE1, GALM, TMSB4X, SOD2, P4HA1, TMSB10, PATL1, TGM2, GNB2, MCFD2, CMBL, TNKS1BP1, GSN, MARCKS, S100A6, FKBP10, FHL2, HRSP12
Downregulated	SEC63, DPH5, NONO, MATR3, DDX5, KIAA1524, MYLK, NHP2L1, RPL29, RPL35, HNRNPR, HSD17B11, CCDC134, HSBP1, MSH2, ACTR1B, PITHD1, EEF1A1, SETD3, PRKDC, DHX9, ELAVL1, RPL14, MPDU1, TBC1D23, RPL21, TAGLN3, TOLLIP, TBC1D13, HLA-A, MYO5A, SACS, UCC1, SF1, ENOPH1, KATNAL2, FBXO7, PPP6C, EIF5B, IFI35, FAM213A, PSME3, RPL8, PMVK, NMD3, EFTUD2, SFPQ, HNRPA1, RPS15, VPS4B, CSNK2A2, SDCBP, PPID, GAPD, IPO9, EIF4A1, ARL1, EIF3M, RPS24, FAU, AHNAK2, HNRNPA3, SAR1A, NDUFAB1, PRSS1, CUL4B, TRIM28, MYO18A, ITGB6, SCAMP3, EIF5, BTF3, HDLBP, SYNCRIP, RPL9, KPNA2, G3BP2, MIF, RPL3, FASN, MCM7, AKR1C3, RPS23, PFDN4, SURF4, EEF1B2, RPL14, DDX39A, ANP32A, AHNAK, RPS25, ABCF2, NME2P1, NAP1L1, RRM1, DDX3X

DEPs: differentially expressed proteins.

**Table 2:** Signaling pathway enrichment analyses of down- and upregulated DEPs.

Pathway ID	Name	Count	*P* value
*Upregulated*			
ko04810	Regulation of actin cytoskeleton	6	9.37*E* − 04
ko05410	Hypertrophic cardiomyopathy (HCM)	3	2.28*E* − 03
ko05412	Arrhythmogenic right ventricular cardiomyopathy (ARVC)	3	2.28*E* − 03
ko04512	ECM-receptor interaction	3	2.28*E* − 03
ko05414	Dilated cardiomyopathy	3	2.28*E* − 03
ko05200	Pathways in cancer	6	2.63*E* − 03
ko05205	Proteoglycans in cancer	5	2.94*E* − 03
ko04151	PI3K-Akt signaling pathway	5	3.30*E* − 03
ko04380	Osteoclast differentiation	3	6.79*E* − 03
ko05032	Morphine addiction	2	6.81*E* − 03
ko04640	Hematopoietic cell lineage	2	6.81*E* − 03
ko04919	Thyroid hormone signaling pathway	3	9.72*E* − 03
ko05020	Prion diseases	2	1.01*E* − 02
ko04727	GABAergic synapse	2	1.20*E* − 02
ko04728	Dopaminergic synapse	3	1.42*E* − 02
ko04145	Phagosome	4	1.43*E* − 02
ko00361	Chlorocyclohexane and chlorobenzene degradation	1	1.46*E* − 02
ko00364	Fluorobenzoate degradation	1	1.46*E* − 02
ko00623	Toluene degradation	1	1.46*E* − 02
ko04510	Focal adhesion	4	1.64*E* − 02
ko05100	Bacterial invasion of epithelial cells	3	2.07*E* − 02
ko04723	Retrograde endocannabinoid signaling	2	2.55*E* − 02
ko04713	Circadian entrainment	2	2.55*E* − 02
ko04130	SNARE interactions in vesicular transport	2	2.81*E* − 02
ko05140	Leishmaniasis	2	2.81*E* − 02
ko04724	Glutamatergic synapse	2	2.81*E* − 02
ko04115	p53 signaling pathway	2	3.07*E* − 02
ko04514	Cell adhesion molecules (CAMs)	2	3.07*E* − 02
ko05133	Pertussis	2	3.63*E* − 02
ko05222	Small cell lung cancer	2	3.63*E* − 02
ko04726	Serotonergic synapse	2	3.63*E* − 02
ko04725	Cholinergic synapse	2	3.63*E* − 02
ko04211	Longevity regulating pathway, mammal	2	3.92*E* − 02
ko05134	Legionellosis	2	4.22*E* − 02
*Downregulated*			
ko03010	Ribosome	13	2.02*E* − 07
ko03008	Ribosome biogenesis in eukaryotes	3	3.27*E* − 02
ko03013	RNA transport	5	4.52*E* − 02
